# Osteoporosis in Inflammatory Arthritides: New Perspective on Pathogenesis and Treatment

**DOI:** 10.3389/fmed.2020.613720

**Published:** 2020-12-01

**Authors:** Denise Rotta, Angelo Fassio, Maurizio Rossini, Alessandro Giollo, Ombretta Viapiana, Giovanni Orsolini, Eugenia Bertoldo, Davide Gatti, Giovanni Adami

**Affiliations:** Rheumatology Unit, University of Verona, Verona, Italy

**Keywords:** osteoporosis, rheumatoid arthritis, psoriatic arthritis, spondyloarthritis, fractures, bone mineral density

## Abstract

Osteoporosis is a skeletal disorder characterized by impaired bone strength and increased risk of fragility fracture and is among the most relevant comorbidities of rheumatic diseases. The purpose of the present review is to discuss the pathogenesis of local and systemic bone involvement in inflammatory arthritides, especially Rheumatoid Arthritis, Psoriatic Arthritis, and Spondyloarthritides, as well as the effect of anti-rheumatic treatments and anti-osteoporotic medication on bone health and fracture incidence, including recent data on novel therapeutic perspective.

## Introduction

Inflammatory arthritides are frequently associated to systemic skeletal complications, such as osteoporosis and fragility fractures. Herein, we presented a review of the available literature on osteoporosis in inflammatory arthritides, with a special focus on Rheumatoid Arthritis (RA), Psoriatic Arthritis (PsA), and Spondyloarthritides (SpA). We also concentrated on the effects of anti-osteoporotic medications on the skeletal involvement in such diseases and on the effects of anti-rheumatic treatments on bone heath and fracture incidence.

## Rheumatoid Arthritis

### Pathogenesis of Osteoporosis in Rheumatoid Arthritis

RA is a chronic systemic disease characterized by pain and joint inflammation with a prominent involvement of the small joints of the hands and feet, leading to joint deformity and functional loss (1).

Due to the presence of a variable grade of systemic inflammation, many comorbidities can accompany the joint involvement, such as extra-articular manifestations and osteoporosis (2–4).

Osteoporosis (OP) is a relevant comorbidity of RA and is caused by the concurrence of several factors such as systemic inflammation, cytokine secretion and circulating autoantibodies. Moreover, the frequent and chronic use of glucocorticoids in RA can also exacerbate the development of OP. Despite adequate strategies in OP primary prevention, such as vitamin D and calcium repletion, most patients will develop OP during their disease history (5).

There are several mechanisms involved in the pathogenesis of OP in RA patients, which is characterized by two main features: local and systemic bone loss (5).

Local bone loss, also called periarticular osteopenia, is characterized by both an impairment of trabecular and cortical bone. Indeed, RA patients have an increased cortical porosity with lower volumetric bone mineral density (vBMD) (6, 7). The cortical thinning has been demonstrated to affect the entire surface of the bone but it is particularly pronounced at the insertion of the synovia, a susceptibility that drives the genesis of bone erosions in RA (5, 8).

Periarticular bone loss, generally preempted by the presence of bone marrow edema (5), appears during an early stage of the disease and is independently associated with the development of bone erosions (9).

The pathogenesis of local bone loss is multifactorial, and many mechanisms contribute to it, such as proinflammatory cytokine secretion (es. IL-6 and TNF) (6, 7), T-cell derived receptor activator of nuclear factor kappa B ligand (RANKL) hyperexpression (10, 11) and a hypothesized direct effect of autoantibodies against citrullinated proteins (ACPA) (7, 12, 13). It was demonstrated that RA patients develop ACPA years before the clinical onset of the arthritis and frequently local bone loss is already present during the preclinical phase of the disease (14, 15). Moreover, the presence of local bone loss at the metacarpal site, detected with High Resolution peripheral Quantitative Computed Tomography (HRpQCT), was observed in RA patients with ACPA positivity, but not in ACPA-negative subjects or in other seronegative inflammatory arthritides such as psoriatic arthritis (PsA) (16). Indeed, ACPA positivity in PsA patients was associated with an erosive form of the disease, strengthening the theory of a direct role played by these antibodies in causing periarticular bone loss (17). This effect might be potentially mediated by a direct stimulation of autoantibodies and osteoclasts FcγR, enhancing osteoclasts maturation (18). In addition macrophages, stimulated by autoantibodies, release inflammatory cytokines which might be able to enhance osteoclast differentiation and function (18).

Osteopenia affects both trabecular and cortical bone, but cortical sites, such as femoral neck and distal radius, seem to be maximally involved (19). Several mechanisms contribute to the detrimental effect on bone homeostasis, overlapping their pathogenetic role on local bone loss.

Inflammatory cytokines such as TNF, IL-6, IL-1, and immune cell-derived RANKL stimulate osteoclastogenesis, while decreasing osteoblastogenesis (20). Moreover, higher levels of circulating senescent CD4+ CD28- T cells were found in RA patients with lower BMD. This subpopulation is known to present a major expression of RANKL compared to CD28+ T cells and to induce osteoclastogenesis in a more efficient way (11).

Furthermore, a subset of patients with RA develops functional antibodies to osteoprotegerin (OPG) and the presence of these antibodies has been associated to higher disease activity and increased bone resorption (21).

Metabolic factors have a central role as well. Dickkopf-related protein 1 (Dkk-1), a Wnt signaling inhibitor, is involved in bone remodeling and OP development (22) and greater serum levels of this inhibitor of bone formation were detected in RA patients compared to healthy controls (23, 24).

As previously discussed, ACPA positivity is an independent risk factor for local bone loss in RA patients, but their action seems to be detrimental also for systemic bone loss. Indeed, a correlation between the low BMD and ACPA serum titer has been demonstrated recently (25).

Systemic and local bone loss are strictly linked and the presence of systemic bone loss, found in almost 60% of early RA patients, is a strong predictor of radiographic joint damage (26). Moreover, a possible association between low systemic BMD and atlantoaxial subluxation occurrence was suggested in subjects affected by RA (27). This evidence suggests that OP could increase susceptibility to bone erosions in RA patients (28, 29).

Chronic treatment with glucocorticoids represents an independent risk factor for the premature onset of OP in RA patients, even if there is still some controversy regarding the safe dosage for bone health. Indeed low-dose and short-term treatment with glucocorticoids in patients with active RA may have a favorable effect by reducing bone resorption driven by systemic inflammation, as seen in many studies and meta-analyses that showed non-significative BMD changes in low-dose glucocorticoid users affected by active RA (30–33). Nevertheless, glucocorticoids have a well-documented detrimental effect on bone homeostasis (34–36) and other studies showed that even low-doses or intra-articular glucocorticoids may have a relevant role in increasing the risk of fracture in RA patients (37, 38).

Not surprisingly, post-menopause is a supplemental risk factor for OP in RA women (39).

### Epidemiology of OP in RA

As previously discussed, RA patients have lower BMD levels at lumbar spine and femoral sites than healthy subjects and this difference can be detected also in an early phase of the disease.

The prevalence of osteoporosis and osteopenia is estimated to be doubled in RA patients as compared to healthy controls (40, 41) with a prevalence ranging from 30 to 50% (40, 42, 43). The risk of developing OP in RA is correlated with the duration and the severity of the disease (19, 44) and even RA pre-menopausal women or men are exposed to a greater risk of osteoporosis in comparison with age- and sex-matched healthy controls (45, 46). A prospective longitudinal study on 379 patients identified some biomarkers predictive of BMD change in patients with RA (47). It was found that the annual BMD change at the lumbar spine had a significant association with glucocorticoids use, bisphosphonate or vitamin D use, and homocysteine. On the other hand, BMD changes at the femur were associated with DAS28, C-reactive protein (CRP), and ACPA titer (47). These results further highlight that there is a strict association between cortical bone health (femoral site) and disease activity while trabecular bone is more affected by classical risk factors (i.e., vitamin D assumption, glucocorticoid use).

Albeit OP is a major problem in the management of RA patients, the percentage of patients receiving calcium and vitamin D supplementation is about 45% and only 5.4% are treated with bisphosphonates (48).

### RA Therapy and OP

Several studies investigated the effect of conventional disease modifying anti-rheumatic drugs (cDMARDs) on bone turnover markers in RA patients (49). Overall, the body of evidence orients toward a decrease in osteoclastic activity induced by cDMARDs, leading to a positive effect on bone mineral density. However, it is not clear if such positive effect corresponds to a fracture risk reduction (50).

Again, controversial results have been found regarding the effect of biological DMARDs (bDMARDs) on BMD and bone markers changes, possibly in relation to the presence of confounding factors such as vitamin D deficiency or corticosteroid therapy (51–56). However, treatment with biologics seemed to be associated with a decrease in bone loss (52).

The first molecules to be studied among bDMARDs were TNF inhibitors, which were associated with a significant, although small, reduction of the vertebral fracture risk in RA patients (57).

A recently published study investigated the effect of TNF inhibitors on BMD and bone biomarkers in patients with RA and ankylosing spondylitis (AS) (58). It was demonstrated that 12 months treatment with anti-TNF drugs prevented further generalized bone loss at both lumbar spine and femoral neck. Moreover, it was found an inverse correlation of baseline C reactive protein (CRP) levels with BMD values, both at baseline measurement and after 12 months treatment, suggesting that baseline high-grade inflammation was associated with lower BMD (58).

IL-6 blocking agents showed an efficacy in reducing systemic bone resorption. As highlighted in a substudy of the OPTION trial, a decrease in bone degradation markers was found in patients treated with tocilizumab, an anti-IL6 agent, plus methotrexate (MTX) as compared to MTX plus placebo (59). In addition, the RADIATE study confirmed such finding in anti-TNF refractory patients (60). Moreover, IL-6 blocking agents were successful in reducing RA localized bone loss, as discussed in a study performed by Axmann et al. (61). Indeed, they found that IL-6 blockade reduced bone erosion in TNF-α transgenic murine models through a direct effect on osteoclast activity which was independent from the anti-inflammatory action (61). Other studies on human subjects further confirmed the positive effect of tocilizumab on bone erosions (62) and systemic bone loss, especially in ACPA positive patients (52, 63).

In 2012 the first Janus Kinases (JAK) Inhibitor was approved for the treatment of RA. The data on the efficacy of these small molecules in reducing systemic bone loss are scarce. A small pilot study showed that tofacitinib, possibly by halting inflammation, can regulate the secretion of serum RANKL and OPG, with a favorable effect on the RANKL/OPG ratio. The interesting speculation of the authors was that tofacitinib could regulate synovial hyperexpression of RANKL via the inhibition of the secretion of IL-17 and IL-6. These effects on RANKL promoted an increase in the osteocalcin levels from 6.9 ± 4.3 ng/mL at the baseline to 8.8 ± 6.1 after 12 weeks of treatment with a concomitant reduction in NTx serum levels (64).

### OP Treatment in RA

Bisphosphonates (BPs) are widely used in post-menopausal OP treatment, because of their attested efficacy in fracture prevention with an acceptable safety profile (65, 66). The majority of data on fracture risk reduction in RA patients of these medications are available only from indirect evidence coming from glucocorticoid induced osteoporosis (GIOP) clinical trials (67). In a 2006 placebo-controlled RCT RA patients on glucocorticoids were randomized to receive placebo or daily doses of alendronate (68). A significant difference was found between the two groups in BMD increase at lumbar spine and femoral site (major in the alendronate group), although without any relevant difference in fracture reduction (68). However, the RCT was not powered to detect any fracture difference.

A recent study explored the effect of zoledronic acid on secondary osteoporosis in 66 RA patients (69). Patients were randomized into three groups: combination-therapy (zoledronic acid and methotrexate), zoledronic acid monotherapy and methotrexate monotherapy and followed over a 12 months period. A significant improvement in lumbar spine and hip BMD within the combination-therapy group was demonstrated.

Another recent study (RISOTTO study) investigated efficacy of sodium Risedronate for glucocorticoid-induced osteoporosis in patients with RA finding a significant increase in lumbar BMD in the Risedronate group compared to the placebo one after 6-month treatment, with no significant difference in femoral neck and total hip BMD (70).

Denosumab is a human monoclonal antibody against RANKL (51, 71).

A recent study showed that denosumab was superior in improving spine and hip BMD to risedronate in GIOP patients, 40% of them affected by RA (72). The 24 months extension of this study, further confirmed the superiority of denosumab over risedronate, albeit no significant differences were found in fracture incidence (73). However, as opposite to the FREEDOM study, which was performed on >7,000 patients followed for 3 years, this GIOP study didn't have an adequate statistical power to detect fracture differences between denosumab and risedronate (an active comparator) (71).

A clinical observational study, recently published, showed the results of denosumab therapy over a 3-years period within two groups of female patients, the former with RA, the latter with primary OP (74). The ΔBMD for the total hip, femoral neck and lumbar spine as well as ΔP1NP did not differ significantly between the two groups at any time points (1, 2, and 3 years assessment), hence, denosumab treatment for osteoporosis had a similar efficacy over 3 years among women with RA and OP.

Another randomized, placebo-controlled study investigated the effects of Denosumab combined with Methotrexate on bone erosion progression in Japanese patients with RA (75, 76). Denosumab significantly inhibited radiographic progression of the disease.

In a recently published study these data have been confirmed (77). This study, named DESIRABLE study, proved the efficacy of denosumab in reducing erosions when added to csDMARD therapy in RA patients. Since there was no improvement in disease activity level, it is reasonable presuming that denosumab action is driven only by a direct effect on bone metabolism rather than an additional indirect way on immune system cells (2). Moreover, denosumab prevented bone erosion but it did not prevent joint space narrowing (77–79), showing its effect in impairing bone destruction but not cartilage destruction. In addition, no differences in infection rate were found between the two groups (77).

Also combined with biological DMARDs (bDMARDs) denosumab was attested to be more efficient in reducing radiographic progression rather than bDMARDs alone (80, 81).

Moreover, switching from bisphosphonates to denosumab significantly reduced radiographic joint destruction compared to continuing bisphosphonates (82). In the same cohort, switching from bisphosphonates to teriparatide was associated with a worse outcome on bone erosions. This result was somehow expected given the detrimental effect of teriparatide on cortical bone health (83).

Romosozumab is a humanized monoclonal antibody that binding to sclerostin neutralizes its blocking effect on Wnt signaling bone formation pathway (84). So romosozumab increases bone formation and, differently from classical osteoanabolic agents, suppresses bone resorption. The role of romosozumab in RA patients or GIOP patients is still unclear. In particular, caution should be taken regarding sclerostin inhibition in RA patients. Indeed, sclerostin inhibition was shown to induce an exaggerated inflammatory response in an arthritis mouse model by promoting TNF secretion (85).

New suggestions for future treatment perspectives have been debated in a very recent review by Gambari et al. which explored the mechanisms regulating monocytes/macrophages fusion and multinucleation (M-FM), a key process to generate mature multinucleated cells such as giant cells (GCs) and osteoclasts (OCs), depending on environment features (86). As discussed in the article, targeting M-FM process in OP therapy might be an interesting alternative to decrease OCs function while preserving osteoblasts (OBs)-OCs communication. In this field, different molecules have been found, like miRNA, that can specifically inhibit M-FM and could be a challenging area to be explored. But targeting the process of monocyte-macrophage differentiation could have interesting implications also for RA therapy strategies. In fact, activated macrophages has an established role in feeding the inflammatory milieu at the synovium site in RA, leading to bone remodeling and erosions; indeed drugs targeting inflammatory cytokines such as TNF, IL-1, and IL-6 have a significative impact on osteoclastogenesis and prevent generalized and local bone loss. A new intriguing opportunity might be to modulate the activation status of macrophages by targeting macrophage fusion and multinucleation, instead of inflammatory cytokines, as a way for preventing an excessive OCs and GCs formation and inflammatory activation (86). In this perspective F-MF inhibition, a mechanism already listed for OP, might be helpful also for preventing bone loss in RA. However, albeit good promises as therapeutic strategy for OP and RA bone loss, clinical trials are mandatory to validate the efficacy, safety and potential superiority of multinucleation inhibitors to current drugs.

## Psoriatic Arthritis and Spondyloarthritis

Psoriatic arthritis (PsA) is an inflammatory musculoskeletal disease commonly associated with psoriasis (PsO), that can be characterized by heterogeneous manifestations, including peripheral arthritis, axial involvement, enthesitis, and dactylitis (87). PsA has an estimated prevalence in the general population of 0.05–0.25%, with little discrepancy depending on the country examined (87, 88). Many comorbidities are associated with PsO/PsA such as increased cardiovascular risk, diabetes, osteoporosis, metabolic syndrome, ophthalmic disease, depression, and anxiety (89).

### Epidemiology of OP in PsA and the Other Spondyloarthritides

Several observational studies have investigated the association between psoriasis or psoriatic arthritis and low BMD values or osteoporosis, with conflicting results.

A cross-sectional study performed on a large population sample with PsO or PsA found that these conditions were significantly associated with osteopenia, osteoporosis, and fragility fractures (90). Moreover, a 2013 population-based analysis showed a significant association between osteoporosis and previous diagnosis of psoriasis in both sexes (91).

On the contrary, a Norwegian study based on hospital-derived fractures data found no association between psoriasis and forearm/hip fracture risk or between psoriasis and osteoporosis (92). Moreover, no differences were found in lumbar spine BMD between Spanish patients with or without PsA (93) and similar findings were underlined during another study conducted in Norway (94).

In 2020, Xia et al. explored the correlation between psoriasis, psoriatic arthritis and osteoporosis considering potential confounding factors that could justify the aforementioned controversial results (95). Conditional regression analyses were performed, and three models were examined: model 0, which included age, height, weight, smoking, and drinking status, model 1, which included all the factors comprised in model 0 plus regular physical activity and model 2, which included all the factors of model 1 plus concomitant medications. In their analyses no estimated bone mineral density (eBMD) difference was highlighted between patients with psoriasis (without arthritis) and healthy controls, therefore psoriasis alone was not correlated to osteopenia and osteoporosis.

More interestingly, it was found that PsA was significantly associated with low eBMD levels even after adjustment for confounding factors in model 0 (age, sex, height, weight, smoking, and drinking status), but this significance became less strong when physical activity was included. Moreover, the association between PsA and low eBMD levels disappeared when conditioning on treatment with methotrexate or ciclosporin (model 2) (95). Therefore, PsA was associated with low eBMD and higher risk of osteopenia, but it seems that this association was somehow mediated by the medical treatment (such as methotrexate or ciclosporin). Furthermore, using a systematic Mendelian Randomization (MR) in order to explore the relationship between PsO/PsA and osteoporosis no causal effects for these factors on BMD score and viceversa were found, reinforcing the idea that the increased risk might be mediated by the treatment and not by the disease itself (95).

However, it's to be noted that all these data refer to quantitative ultrasound (QUS) estimated BMD at heel site, which has high specificity but whose sensitivity to predict BMD as defined by dual-energy x-ray absorptiometry (DEXA) can be significantly variable depending on QUS parameters.

A recently published study compared the incidence of fragility fractures in patients with PsA matched with controls by age and sex (96) and no differences were found in the overall fracture incidence rate. This result was true even for vertebral fractures, that were apparently more frequent in PsA patients compared to controls, but this difference did not reach the statistical significance (96).

Beyond PsA, data on the other Spondyloarthritides (SpA) with axial involvement (axSpA), such as Ankylosing Spondylitis (AS), are heterogeneous (51). Moreover, in patients with axSpA the proper evaluation of vertebral deformities and BMD levels is frequently altered by the presence of syndesmophytes and periosteal bone proliferation (97).

Nevertheless, several data confirm that axSpA patients have lower BMD levels as compared to healthy controls even in the early stage of the disease, independently from spine mobility and exercise (98, 99). The mechanism involved in this process is, at least in part, related to the prevalent inflammation, which can contribute to local bone loss especially in those sites interested by Bone Marrow Edema (BME)/osteitis (100, 101). In fact, inflammation leads to trabecular bone loss, which is associated with the presence of lesions defined as BME/osteitis on MRI that quintuplicate the risk of finding low spine BMD. Hence, the presence of inflammation signs on MRI represents the main risk factor associated with low BMD in axSpA.

Interestingly, the lumbar spine seemed to be more susceptible to bone loss in patient with non-radiographic axSpA (nr-axSpA) as compared to the femur (102).

In addition, the estimated prevalence of osteopenia and osteoporosis within 10 years from the onset of AS is about 51–54% and 13–16%, respectively (99).

Results from a primary care-based nested case-control study showed that AS patients had a higher risk of clinical vertebral fractures without an increased risk of non-vertebral fractures, further highlighting that AS is a risk factor for fractures, but limited to the axial skeleton (103).

In order to find more effective methods to predict the risk of fragility fractures in AS patients, a recent study investigated the usefulness of the trabecular bone score (TBS) in assessing bone strength in patients with AS compared with DEXA (104). TBS showed promising results in improving the ability to detect patients at high risk of fractures, especially in patients with normal or osteopenic BMD levels at standard DEXA (104).

Another recent study investigated the effect of vertebral ankylosis on scanographic bone attenuation coefficient (SBAC), measured from L1 to L5, in AS patients (105). It was found that patients with at least one bony bridge had lower SBAC values, while there was a correlation between the presence of full ankylosis and the probability of presenting SBAC ≤145 HU (fracture threshold), suggesting an impact of ankylosed vertebrae on trabecular bone deterioration.

### Bone Remodeling in PsA and the Other Spondyloarthritides

In contrast to RA, PsA, and the other SpA, such as AS, are characterized by the simultaneous occurrence of both bone resorption and bone formation signs, the latter of which has not been observed in patients with RA (106).

While most of the mechanisms leading to bone erosions are well-understood and overlap those in RA (107), part of the mechanisms of bone formation in SpA remain unknown. Pro-inflammatory cytokines (such as TNF-α and IL-17) and signaling pathway (including Wnt pathway) have been shown to be involved (108). IL-17, the prevailing inflammatory cytokine in many patients with SpA (109), has been shown to promote both OC bone resorption and OB bone formation (109), while TNFα has a greater effect on promoting bone erosion and suppressing OBs function. Entheses, are poorly populated by OCs and when reached by the IL-17 inflammatory stimulus, bone formation may be induced in a way that can overcome bone resorption (109).

Nevertheless, despite several data, many features of this process remain still incompletely clarified.

IL-23 is another distinctive cytokine that is abundantly present in the affected tissues of PsA patients. IL-23 is generally overexpressed in PsA, leading to an upregulation of IL-22 and consequently to osteoblast-related genes induction, which eventually results in osteoblast expansion and enthesophytes formation (110). Moreover, a recent study exploring Wnt pathway regulators found significantly lower levels of Dkk-1 in patients with PsA rather than RA or healthy controls, suggesting another possible explanation for the different bone phenotype between RA (erosive) and PsA (erosive-proliferative) (111). Furthermore, possibly in relation to a negative feedback response, a rapid increase in Dkk-1 after 6 months of secukinumab, an anti-IL17 antibody, was recently observed (112). This finding might well-explain the promising results that IL-17 blockade had achieved in halting the syndesmophytes progression in AS. Indeed, in this setting, the raise of Dkk-1 might be even beneficial by stopping the bone proliferation induced by the Wnt pathway.

Moreover, a dysregulation in RANK-RANKL pathway had been reported in patients with AS. Increased expression of intracellular RANKL levels in CD4+ and CD8+ T cells and a significantly lower expression of membrane-bound RANKL were found in AS patients (113).

A 2016 study explored the association of Dkk-1 levels with low BMD and vertebral fracture prevalence among patients with AS (114). AS patients had lower levels of Dkk-1 compared to healthy controls; however Dkk-1 serum levels inversely correlated with lumbar spine Z-score BMD and higher serum levels of Dkk-1 were associated with a higher prevalence of 1 or more vertebral fractures. So serum Dkk-1 titer, even though lower in AS patients, than in healthy controls, appears to be associated with an increased risk of severe osteoporosis (114).

Furthermore, a correlation between Dkk-1 and PTH was observed, with higher levels of PTH and lower levels of Dkk-1 measured in AS patients rather than healthy controls (115). Then dividing the patients in two equal groups according to disease duration, the association between PTH and Dkk-1 remained only in the group with longer disease duration, where Dkk-1 levels were also correlated with higher CTX and lower BMD (Z-score ≤ – 1) (115).

In addition anti-OPG antibodies has been isolated in a cohort of SpA patients and a correlation with low BMD and fractures was found (116).

In summary, multiple players are involved in bone remodeling of PSA/SpA, with a combination of systemic effects mainly driven by inflammatory cytokines and metabolic factors.

### SpA Therapy and OP

Non-steroidal anti-inflammatory drugs (NSAIDs) play a pivotal role in the treatment of SpA, especially in those with an axial involvement (axSpA), in whom NSAIDs represent the first line treatment (117). However, despite their wide use, few data have been collected about their possible effects on bone (51). A case-control study found a decreased risk of any clinical fracture in patients with AS treated with NSAIDs (103). Moreover, data from a large population-based public health database supported the protective role of NSAIDs on clinical fracture risk in SpA patients, highlighting a higher fracture risk in those not assuming chronic NSAIDs (118).

TNF inhibitors use was associated with an increase in lumbar spine and total hip BMD and maintenance of femoral neck BMD for up to 2 years in patients with AS (119). Another study investigated the effects of 4-years course with anti-TNFs in patients with long-term AS and albeit no differences were found in the incidence of fragility fractures the authors found an improvement in BMD levels during 4 years period (120).

Moreover, a single-center study explored the effect of the TNF-blocker infliximab vs. i.v. neridronate (a potent amino-bisphosphonate) on bone turnover and disease activity after 3–6 months treatment in AS patients (121). As regards bone metabolism, no significant BMD variations were observed at 6 months in the infliximab group, while a significant BMD increase at LS site, was found in the bisphosphonate arm (p<0.05 vs baseline and vs infliximab) (121). In addition i.v. neridronate was as effective as infliximab in controlling the leading symptoms (as assessed by BASDAI or BASFI) of AS even without, as expected, any changes in systemic inflammation parameter (CRP and ESR), suggesting that, at least in part, the symptoms of AS are related to bone metabolic factors.

In fact, as known (122), bisphosphonates reduce bone resorption, which is elevated mainly in the early stages of AS. Furthermore, through the coupling of osteoclast and osteoblast activity, this mechanisms leads to later inhibition also of bone formation and causes an increase in serum sclerostin, which is low in AS and negatively correlated to the development of syndesmophytes (123, 124).

As concerning PsA, a recent study compared the effects of methotrexate (MTX) with bDMARDs on bone structure and biomechanical properties (125). It was found that bDMARD-treated patients had higher bone mass and better bone strength than patients receiving MTX or no DMARDs (despite longer disease duration in bDMARDs-treated group) (125).

JAK-inhibitors had been recently approved for the treatment of SpA. While a reduction in articular bone erosion in RA and PsA patients has been shown during treatment with tofacitinib, still no data on BMD or osteoporosis has been published (110).

## Conclusions

OP is a hallmark of inflammatory arthritides. Its pathogenesis is manly driven by the predominant inflammation, notwithstanding that, other metabolic factors are increasingly believed to play a crucial role in the development of OP and fragility fractures in such diseases. Novel therapeutic agents have been approved for the treatment of inflammatory arthritides and their role in preventing or even treating osteoporosis is becoming clearer.

**Figure 1:**
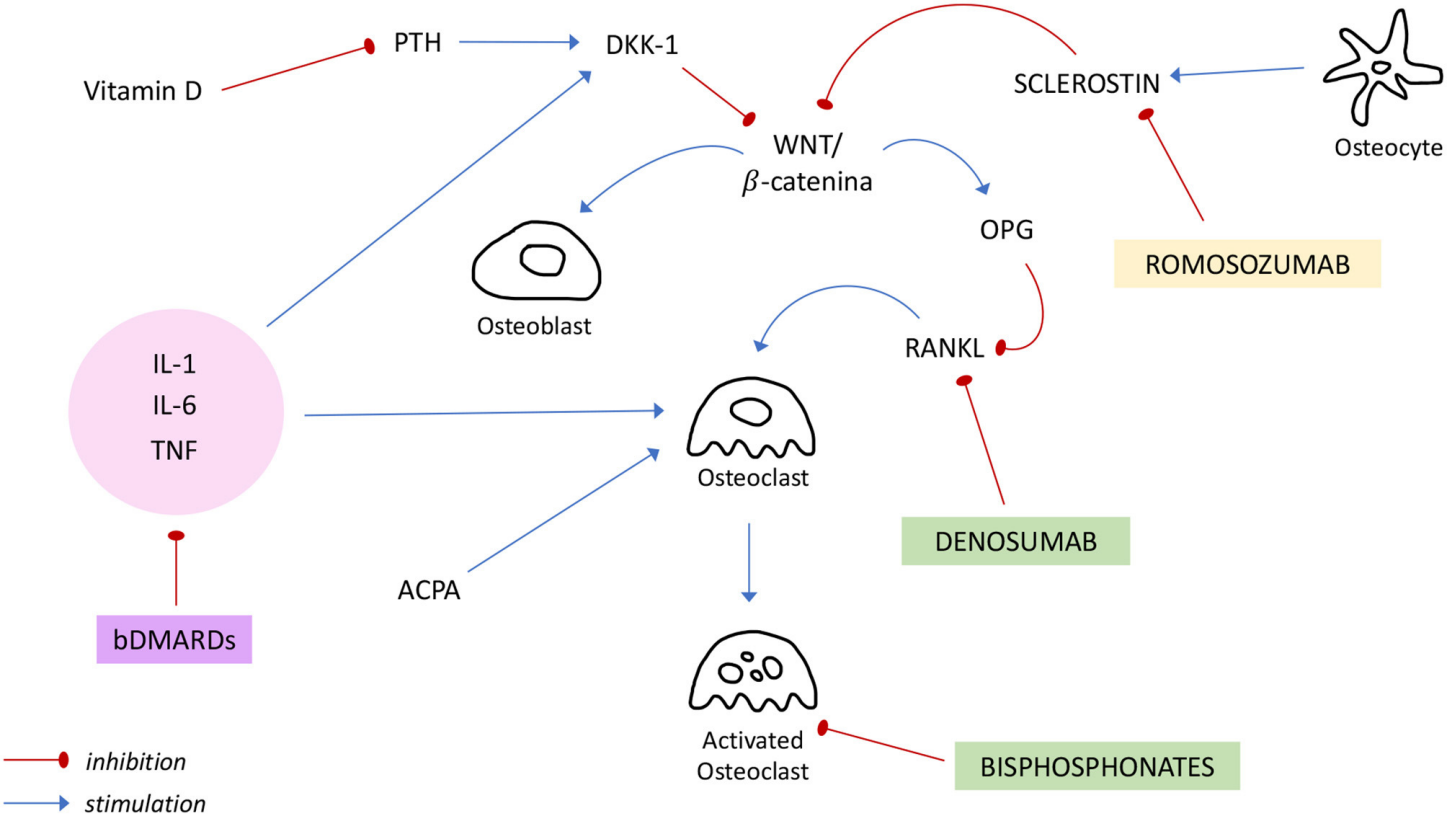
Regulation of bone metabolism and mechanisms of action of bDMARDs and anti-osteoporotic drugs. *ACPA*, anti-citrullinated protein antibody; *bDMARDs*, biologic Disease Modifying Anti Rheumatic Drugs; *DKK-1*, Dickkopf-1; *IL*, interleukin; *OPG*, osteoprotegerin; *PTH*, Parathormon; *RANKL*, receptor activator of NF-κB ligand; *TNF*, tumor necrosis factor.

In the figure above (Figure 1) the principal targets of bDMARDs and anti-osteoporotic drugs previously discussed are summarized.

Nevertheless, albeit clinicians can benefit from new treatment discovery, further research is needed to expand our knowledge about the interaction between bone metabolism and inflammation, in order to find shared mechanisms to target and expand the therapeutic armamentarium available in the clinical practice either to prevent or to treat the bone loss in arthritides.

## Author Contributions

All authors listed have made a substantial, direct and intellectual contribution to the work, and approved it for publication.

## Conflict of Interest

The authors declare that the research was conducted in the absence of any commercial or financial relationships that could be construed as a potential conflict of interest.
